# The Study of Thermal, Mechanical and Shape Memory Properties of Chopped Carbon Fiber-Reinforced TPI Shape Memory Polymer Composites

**DOI:** 10.3390/polym9110594

**Published:** 2017-11-10

**Authors:** Zhenqing Wang, Jingbiao Liu, Jianming Guo, Xiaoyu Sun, Lidan Xu

**Affiliations:** College of Aerospace and Civil Engineering, Harbin Engineering University, Harbin 150001, China; wangzhenqing@hrbeu.edu.cn (Z.W.); liujingbiao1213@163.com (J.L.); guojianming1218@163.com (J.G.); xulidan0501@126.com (L.X.)

**Keywords:** polymer composite, mechanical properties, shape memory properties, thermodynamics

## Abstract

Trans-l,4-polyisoprene (TPI) shape memory polymer composites with different chopped carbon fiber mass fractions were prepared to study the effects of different chopped carbon fiber mass fractions and temperatures on the TPI shape memory polymer composites in this paper. While guaranteeing the shape memory effect of TPI shape memory polymers, the carbon fiber fillers also significantly enhanced the mechanical properties of the polymers. The thermodynamic properties and shape memory properties of TPI shape memory polymers were studied by a differential scanning calorimeter (DSC) test, dynamic mechanical analysis (DMA) test, thermal conductivity test, static tensile test, mechanical cycle test, thermodynamic cycling test and shape memory test. Furthermore, the tensile fracture interface of TPI shape memory polymer composites was analyzed by scanning electron microscopy. The experimental results show that when the chopped carbon mass fraction fiber is 8%, TPI shape memory polymers have good shape memory properties and the best mechanical properties.

## 1. Introduce

As a new smart material having broad application space, according to differences of recovery stimulation, shape memory polymers (SMPs) can be divided into the following groups: thermo-responsive SMPs, chemo-responsive SMPs and photo-responsive SMPs [1,2,3]. Compared with shape memory alloys (SMAs), SMPs have done well in many aspects such as light weight, large recovery ability [4], excellent molding ability [5] and low cost [6]. In recent years, SMPs have been under development in some new fields such as functional clothing [7], information carriers [8,9], two-way actuators [10,11,12] and active assembly/disassembly [13]. Although there is a wide variety of SMPs, thermo-responsive SMPs are still the most basic and the most common SMP materials [14,15,16]. 

**Figure 1:**
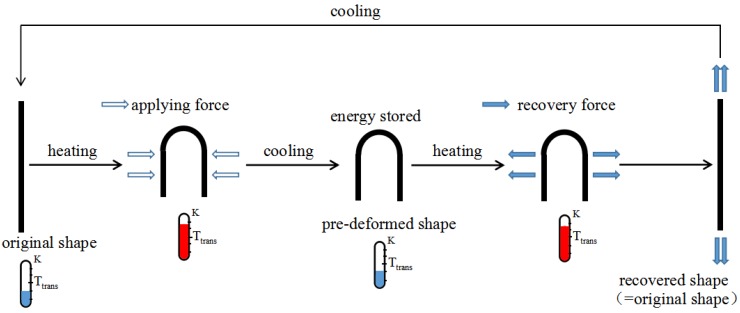
Schematic of thermomechanical deformation process of shape memory polymers (SMPs).

**Figure 2:**
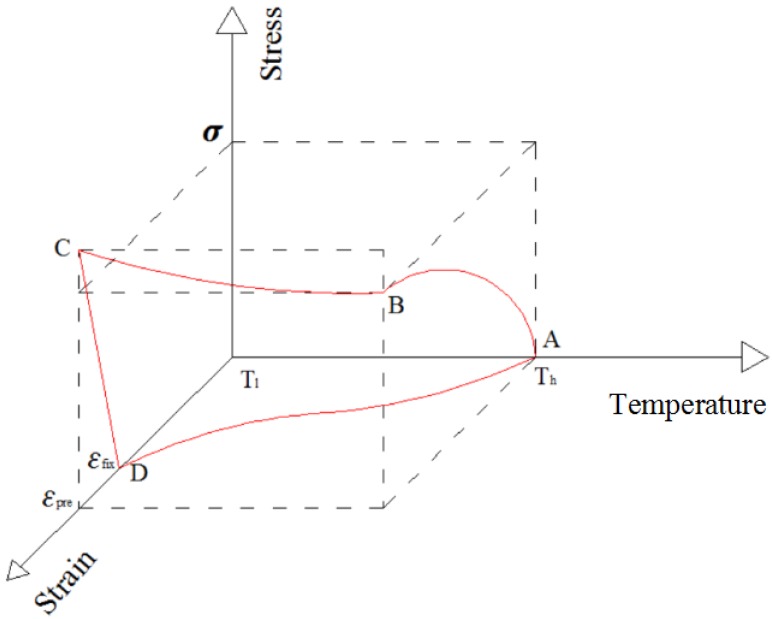
3D stress–strain–temperature diagram illustrating the thermomechanical behavior of SMPs.

The thermomechanical deformation process of SMPs is schematically shown in Figure 1. The thermomechanical deformation process of SMPs consists of the following steps: (1) heat and deform SMP above the transition temperature $T_{trans}$; (2) cool SMP until below $T_{trans}$ and remove the applying force; (3) heat the pre-deformed SMP above $T_{trans}$ and then it will recover to its original shape; (4) cool SMP below $T_{trans}$ with its original shape kept. A 3D stress–strain–temperature diagram illustrating the thermomechanical behavior of SMPs is shown in Figure 2 and the thermomechanical behavior of SMPs can be illustrated in four steps [17,18,19]. The first step (state A→B) is a high-temperature deformation state. At this step, the elastic modulus of the SMP is low, the fracture elongation is large, and some of the SMP material shows obvious viscoelastic properties. The second step (state B→C) is a constant-strain cooling state. With a decrease of temperature, the elastic modulus of SMP increases gradually, and the stress required to maintain the constant strain also increases gradually. The third step (state C→D) is a low-temperature unloading state. When the stress is unloaded to zero, in addition to partial strain recovery, most of the pre-strain is stored in the SMP in the form of storage strain. The fourth step (state D→A) is a heating-up recovery state. The stress is zero at this step, and the pre-strain decreases gradually with an increase of temperature until the SMP achieves a full recovery. 

Trans-l,4-polyisoprene (TPI) is a high-performance new synthetic rubber material with low dynamic heat build-up, rolling resistance and excellent wear-resistance and dynamic fatigue properties [20]. Compared with other SMPs, TPI SMPs have unique performance advantages, such as more than 200% large deformation recovery capacity and easily-tailored transition temperature ($T_{t}$) around room temperature, about 317–325 K; these values can be compared to shape memory epoxy, epoxy shape memory foams and shape memory cyanate polymers, whose respective values of fracture strain are 27%, 90% and 3%, and whose respective $T_{trans}$ values are 328 K, 358 K and 485 K [21,22,23]. However, the weakness of the mechanical properties of TPI SMPs constrains their application to a great extent [24,25]. It is important to find processing methods of enhancing TPI’s mechanical performance, and adding functional fillers is the most direct method to improve its mechanical characteristics. Some concepts have been successfully realized by adding functional fillers to SMPs. For example, the properties of polyurethane shape memory polymer composites (SMPCs) were studied by adding silica nanofiller to polyurethane SMP, and an ideal result with respect to crystallization kinetics was achieved [26]; furthermore, cellulose whiskers as filler were added into an epoxy polymer, and a small amount of cellulose whiskers with good dispersion could effectively improve the stiffness of the SMP, and had no negative effect on the shape memory effect of the SMP [27]. Therefore, TPI shape memory polymer composites (TPI SMPCs) are developed by adding reinforcing fillers to TPI SMPs [28,29,30]. While guaranteeing the shape memory effect of TPI SMPs, this method can also significantly enhance their mechanical properties. 

The mechanical properties and shape memory performance of SMPs are enhanced by physical blending [31], in-situ polymerization [32], chemical crosslinking and so on [33]. According to the types of reinforcing filler, SMPCs can be broadly divided into three categories: nanomaterial-reinforced composites, particle/short fiber-reinforced composites and continuous fiber-reinforced composites [34,35,36,37]. Among them, fiber-reinforced SMPCs are mainly used as structural materials because of their better comprehensive mechanical properties [38], larger recovery force [39], and higher structure stiffness and strength [40]. Therefore, this paper studies chopped carbon fiber-reinforced TPI SMPCs to improve the mechanical properties and shape memory properties of TPI SMPs.

## 2. Experimental

### 2.1. Materials and Preparation

In the preparation process of TPI SMPCs, the main raw materials formulation is shown in Table 1. The matrix used in this paper was TPI, which was produced by Shanghai Shucan Industry Co., Ltd. (Shanghai, China). The reinforced material used in this paper was chopped carbon fiber with an average length of 2 mm, produced by Shanghai Staple Fiber Co., Ltd. (Shanghai, China), and the mass fraction of chopped carbon fiber was 0%, 4%, 6%, 8%, 10%, 12% or 14%.

**Figure 3:**
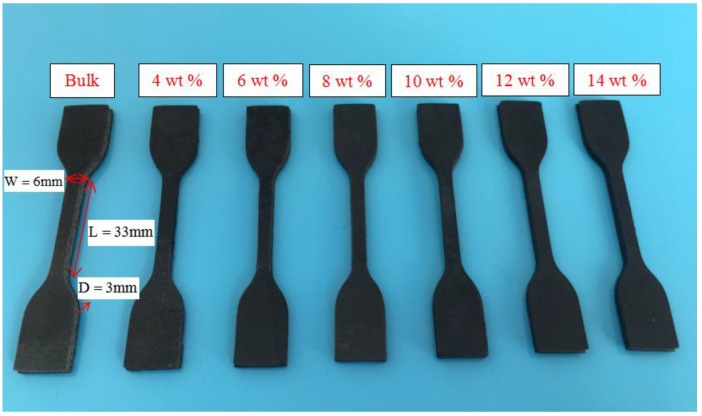
TPI SMPC specimens.

Some raw materials need to be pretreated before preparing the TPI SMPCs. First of all, we placed TPI in a vacuum drying oven (DZF-6210, Shanghai Yi Heng Scientific Instrument Co., Ltd., Shanghai, China) for 24 h at room temperature. We then added carbon fiber to an acetone solution, and conducted ultrasonic treatment for 10 min, rinsed with distilled water, dried and set aside. A suitable amount of concentrated nitric acid was measured into a flask with three necks, heated up to 300 K, connected to the condensation water, and magnetically stirred; then, we removed the carbon fiber after refluxing for 1 h, 2 h or 3 h. Distilled water and anhydrous ethanol were used to clean the carbon fiber until the pH value was greater than 6, at which point it was dried and set aside. In an open mixing machine (Po Wheel Precision Detection Equipment Co., Ltd., Dongguan, China), we put the surface-treated carbon fiber into TPI with common monomer charge order (adding sulfur and carbon fiber first), mixed and took down-orientation. We then measured the optimum cure time $t_{c90}$ of mixing rubber sample under vulcanization temperature. The mixed raw materials were vacuum treated before hot pressing, and then vulcanization molding was conducted on the Electric Flat vulcanizing machine (Shenzhen Jia Xin Electronic Equipment Technology Co., Ltd., Shenzhen, China). Curing conditions: The curing pressure was 10 MPa and curing temperature was 423 K. After the TPI SMPCs were removed and cooled at room temperature, dumbbell specimens were prepared by using a laser cutting machine (HSLC.1410, Boye Laser Applied Technology Co., Ltd., Jiaxing, China). The effective thickness of the TPI specimen was 3 mm, length was 33 mm, width was 6 mm, and the preparation of the specimen was based on the ASTM D412 standard. The dumbbell specimens are shown in Figure 3.

### 2.2. Properties Test of Shape Memory Polymer/Chopped Carbon Fiber Composites

#### 2.2.1. Differential Scanning Calorimeter (DSC) Test

The study in this paper tested the crystallization and melting transition properties of TPI SMPCs by DSC (STA449, Selbe, Germany). Test temperature range was from 299 K to 339 K, while the variable temperature rate was 3 K/min. The sample mass was about 30 mg, and protection gas was nitrogen.

#### 2.2.2. Dynamic Mechanical Analysis (DMA) Test

Dynamic mechanical properties were measured by a Q800 dynamic mechanical analyzer (American TA Company, San Francisco, CA, USA) to obtain the storage modulus (E′) and loss tangent (tan δ) versus temperature curves. Specimens were scanned from 200 to 300 K, at a frequency of 10 Hz, a maximum amplitude of 120 µm and a heating rate of 3 K/min.

#### 2.2.3. Thermal Conductivity Test

The test was carried out on a NETZSCH laser-guided calorimeter (NETZSCH Scientific Instruments Trading Ltd., Freiburg, Germany) to measure the thermal conductivity of TPI SMPCs at temperatures of 299 K, 319 K and 339 K. The experimental principle of the method was as below: after graphite was uniformly sprayed onto specimens with upper and lower surfaces in parallel, the upper surface received a specific laser pulse wave; at the same time, the temperature response of the lower surface was measured and recorded. According to the mathematical model of the steady heat conduction process, we calculated the thermal diffusivity coefficient α(T) of the specimens at different temperatures, as shown in Formula (1). Then, the thermal conductivity λ(T) at different temperatures was calculated by the conversion relation as shown in Formula (2).

(1) $$\alpha \left(T\right)=0.1388\times d^{2}/t_{50}$$

(2) $$\lambda \left(T\right)=\alpha \left(T\right)\times C_{p}\left(T\right)\times \rho \left(T\right)$$

In Formulas (1) and (2): d is the thickness of specimen, $t_{50}$ is the time needed for the upper surface temperature of the specimen to rise to half of the maximum value after receiving light pulse irradiation, $C_{p}\left(T\right)$ is the specific heat of the specimen at temperature of *T*, and ρ(T) is the density of the specimen at temperature of *T*.

#### 2.2.4. Mechanical and Thermal–Mechanical Properties Tests

The static mechanical property test, mechanical cycle property test and thermal mechanical cycle property test were carried out on a universal testing machine (Instron 4501, Bremen, Germany) to study the mechanical and thermal–mechanical properties of TPI SMPCs. The thickness of the dumbbell specimen being tested was 3 mm, the effective length was 33 mm and the width was 6 mm. The gauge length in all tests was set to 20 mm. The relative crosshead movement was measured and then divided by the gauge length to estimate the strain.

##### Static Mechanical Property Test

The static tensile test was carried out at the crosshead speed of 30 mm/min at different temperatures of 299 K (Tt − 20K), 319 K (Tt) and 339 K (Tt + 20 K). The dumbbell specimens of TPI SMPCs were stretched at constant speed until complete failure, and the engineering stress and engineering strain were used to describe the static mechanical properties of TPI SMPCs.

##### Mechanical Cycle Property Test

The cyclic tension test adopted the loading mode of equal displacement and constant rate (30 mm/min) at room temperature. According to test definitions, the upper limit strain value was set to be 100% and the number of cycles was 5. The stress and strain in this paper are the engineering stress and engineering strain definitions.

##### Thermal–Mechanical Cycle Property Test

To investigate the influence of polymer transition temperature on the mechanical properties of different TPI SMPC systems, thermal–mechanical cyclic tensile tests of TPI SMPCs with different carbon fiber mass fractions were carried out. The test was performed at temperatures of 299 K, 319 K and 339 K, and the crosshead speed was 20 mm/min. The limit of constant strain was 100% and the cyclic number was 5. The stress and strain in this paper adopted engineering stress and engineering strain definitions.

#### 2.2.5. Shape Memory Properties Test

The traditional shape memory test was accomplished by a uniaxial tension or compression test, and the shape memory properties of SMPs were calculated by a using tensile or compressive stress–strain curve. However, the SMP/carbon fiber composites are commonly used for smart skins of advanced aircraft and space-deployable structures of spacecraft, due to their bend and fold characteristics. The testing method of traditional shape memory properties cannot meet their actual needs. Therefore, this paper uses a new method, dubbed the “folding-deploy shape memory test”, to characterize the shape memory properties of TPI SMPCs.

The process of the “folding-deploy shape memory test” included three steps. In the first step, the specimen was heated up to 349 K, which was higher than the transition temperature $T_{t}$; it was then bended and folded into a U shape around a central shaft with a diameter of 4 mm, and the maximum bending angle, $\theta_{\max}$, was recorded In the second step, the specimen was rapidly cooled, and the external force was removed after the shape of the specimen was maintained for some time. A marginal elastic recovery of the specimen would appear, and the fixed angle of the specimen, $\theta_{\mathrm{fixed}}$, was recorded. In the third step, the specimen was reheated to $T_{i}$ to recover its original shape. The temperature was kept for 5 min at each 5 K increment from 299 K to 349 K, and the corresponding bending angle $\theta_{i}$ and the test time *t* of each round of the process were recorded at each temperature.

(3) $$\mathrm{Shape}\;\mathrm{retention}\;\mathrm{ratio:}\;R_{f}=\frac{\theta_{\mathrm{fixed}}}{\theta_{\max}}\times 100\%$$

(4) $$\mathrm{Shape}\;\mathrm{recovery}\;\mathrm{ratio:}\;R_{r}=\frac{\theta_{\max}-\theta_{i}}{\theta_{\max}}\times 100\%$$

(5) $$\mathrm{Shape}\;\mathrm{recovery}\;\mathrm{rate:}\;V_{r}=\frac{\pi (\theta_{\mathrm{fixed}}-\theta_{i})}{180t}$$

## 3. Results and Discussion

### 3.1. DSC

**Figure 4:**
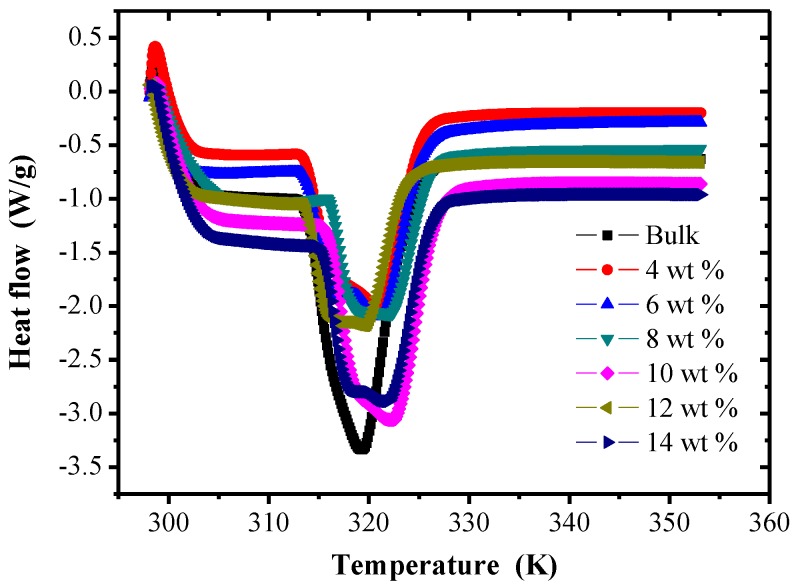
Differential scanning calorimeter (DSC) thermogram of chopped carbon fiber reinforced TPI shape memory polymer composites with different mass fractions.

The DSC thermogram of chopped carbon fiber-reinforced TPI SMP composites with different mass fractions is shown in Figure 4. From the figure, for the bulk system, there is an obvious peak value at a temperature of 319 K, which indicates that there is a state transition inside TPI SMPs at that temperature. Transformation occurs in the heating process, which shows 319 K is the crystallization melting temperature of TPI SMPs. With an increase of chopped carbon fiber mass fraction, the crystallization melting temperature of hard chains of carbon fiber-reinforced TPI SMP composites is increased, compared with the TPI SMP matrix. That means the heat resistance of polymer composites is much better than that of the bulk specimen. In addition, the improvement of the crystallization melting temperature of the hard chains indicates that the micro-phase separation degree of polymer composites is improved. Because the mechanical properties are closely related to the micro-phase separation degree, the higher the micro-phase separation degree, the better the mechanical properties of the polymer composites.

### 3.2. DMA

**Figure 5:**
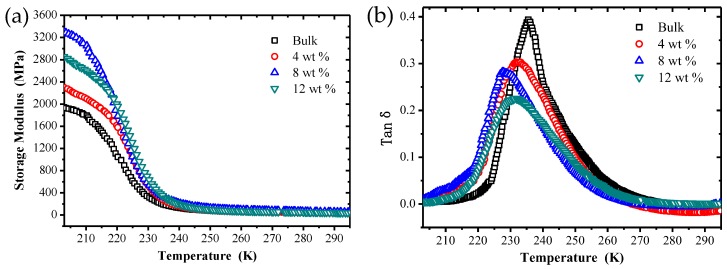
The storage modulus (**a**) and loss tangent (**b**) versus temperature curves of SMPC specimens with different carbon fiber content.

The storage modulus (E′) and loss tangent (tan δ) versus temperature curves obtained from the dynamic mechanical analysis are illustrated in Figure 5. From the Figure 5a, it can be seen that the storage modulus of TPI SMPCs are obviously improved by filling with chopped carbon fiber at different temperatures. When the carbon fiber mass fraction is 4% and 8%, the storage modulus of TPI SMPCs increases with the increased fiber content. This is because the addition of carbon fibers enhances the interfacial stress transfer and improves the rigidity of TPI SMPCs. It is noteworthy that when the carbon fiber mass fraction was 12%, the composite’s storage modulus decreased. This may be because when the carbon fiber content reaches a certain amount, the dispersion of carbon fiber in the polymer matrix gets worse, which causes a large degree of agglomeration, leading to a weak interface layer between the fiber and TPI; the stress transfer effect is reduced, which leads to a decrease of the storage modulus of the SMPC. According to the Figure 5b, it can be clearly observed that the glass transition temperature ($T_{g}$) of the bulk system is 235 K, and with an increase of the carbon fiber mass fraction, $T_{g}$ moves to a lower temperature, which indicates that the chain motion between polymer and filler is enhanced. In addition, the loss factors of TPI SMPCs decrease with an increase of chopped fiber mass fraction, and are lower than the loss factor of the bulk.

### 3.3. Thermal Conductivity

**Figure 6:**
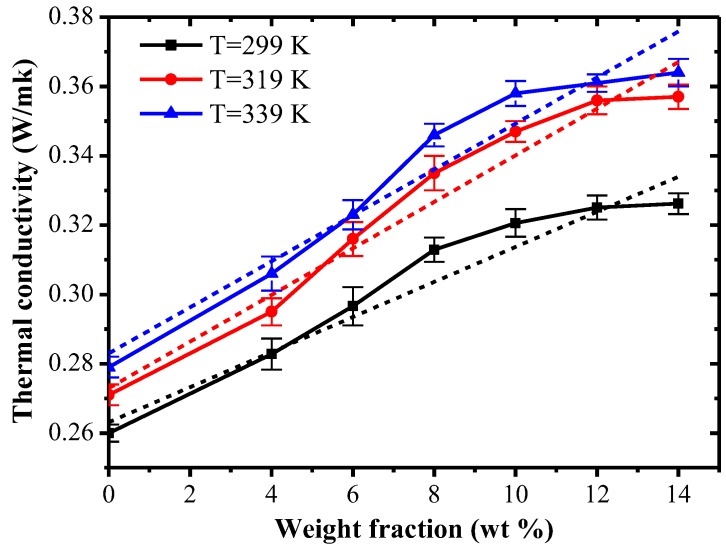
Thermal conductivity of composite systems with different carbon fiber contents at different temperatures.

Figure 6 shows the thermal conductivity of composite systems with different carbon fiber mass fractions under different temperatures. Due to the inconsistency of thermal conductivity of polymer composites at different temperatures, the thermal conductivity of polymer composites were measured at different temperatures of 299 K, 319 K and 339 K.

From Figure 6, with an increase of the carbon fiber content, the thermal conductivity of composites roughly shows a tendency of linear growth. When the mass fraction of carbon fiber is increased from 0% to 14%, the thermal conductivity of composites is respectively increased by 28%, 34% and 36% at temperatures of 299 K, 319 K and 339 K. When the chopped carbon fiber mass fraction is lower than 10%, the increased range of thermal conductivity of the composites is bigger. The reason is that, after chopped carbon fibers with excellent thermal conduction performance are filled into the TPI polymer, when the filling amount reaches a critical value, the filler begins to increasingly form a continuous thermal conduction network chain in the TPI polymer. With the increasing amount of filler, filler is stacked more densely, which makes the heat-flow path strengthen. At the same time, carbon fibers with high thermal conductivity play a main role in the thermal conductivity of composites, and the increased range of thermal conductivity is larger. When the carbon fiber dosage continues to increase, the enhanced range of thermal conductivity of the composite is decreased. The reason may be that the surface of the carbon fiber is smooth, which causes poor adhesion between carbon fiber and the TPI polymer. When the amount of carbon fiber is excessive, the dispersibility of carbon fiber in the TPI polymer gets worse, producing a large degree of agglomeration. Then, incrementing carbon fiber content has little contribution to the heat-conductive network chain, and therefore, the increased range of thermal conductivity of the composite is decreased.

### 3.4. Mechanical and Thermal Mechanical Properties

#### 3.4.1. Static Mechanical Property

**Figure 7:**
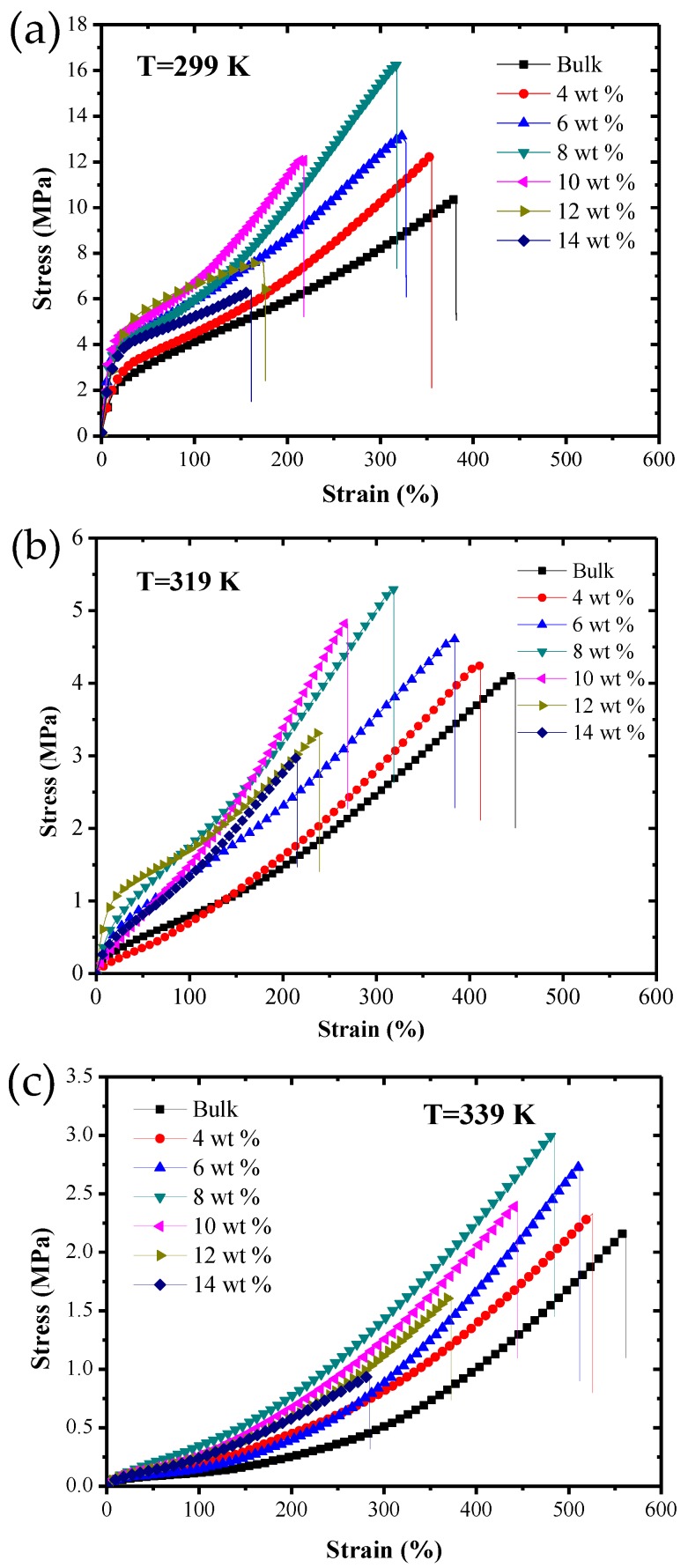
The static tensile stress–strain curves of shape memory polymer composite specimens with different chopped carbon fiber contents at three test temperatures: (**a**) 299 K, (**b**) 319 K and (**c**) 339 K.

Figure 7 shows the static tensile stress–strain curves of SMPC specimens with different chopped carbon fiber contents at three test temperatures: (a) 299 K, (b) 319 K and (c) 339 K. At a temperature of 299 K, the strains of specimens with carbon fiber mass fractions of 0%, 4%, 6% and 8% were all more than 300%. When the chopped carbon fiber mass fraction was more than 8%, the strain value of the specimen significantly decreased. Yield and uniform elongation phenomena can be observed in all specimens except the specimens with carbon fiber mass fractions of 12% and 14%. The fracture stresses were all more than 12 MPa for the specimens with carbon fiber mass fractions of 0%, 4%, 6%, 8% and 10%, and with a continuous increase of the mass fraction, the fracture stress decreased obviously. At a temperature of 319 K, the fracture strains of all specimens increased significantly, while the fracture strain of the bulk was even greater than 450%. The specimens with carbon fiber mass fractions lower than 10% still exhibited higher fracture stress, and the range of fracture stresses was from 4 MPa to 6 MPa. At a temperature of 339 K, the fracture strains of all specimens were greatly increased, while the corresponding fracture stresses decreased significantly, all being lower than 3.1 MPa. Among them, the fracture stress of the specimen with a carbon fiber mass fraction of 14% reduced to only 0.94 MPa.

**Figure 8:**
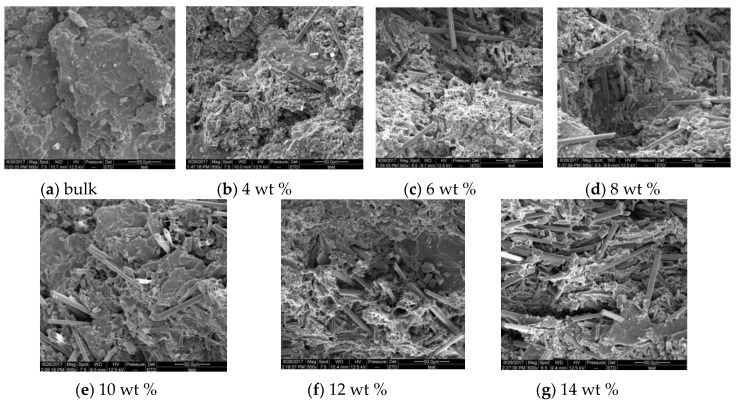
Scanning electron microscopy (SEM) images of cross-sections of SMPC specimens with different chopped carbon fiber contents of (**a**) bulk, (**b**) 4 wt %, (**c**) 6 wt %, (**d**) 8 wt %, (**e**) 10 wt %, (**f**) 12 wt % and (**g**) 14 wt % after static tensile test at a temperature of 299 K.

Figure 8 shows the scanning electron microscopy (SEM) images of cross-sections of SMPC specimens with different chopped carbon content after static tensile test at a temperature of 299 K. It is shown that the cross-section of the bulk is relatively smooth and continuous, presenting a morphology like a beach. When the mass fraction of carbon fiber is 4%, the specimen’s cross-section is rougher compared with the bulk specimen, and has obvious prominences and different degrees of depression. The cross-sections of specimens with carbon fiber mass fractions of 6% and 8% are coarser than the bulk and the 4% specimen. Additionally, the phenomenon that carbon fibers are pulled out from the polymer matrix occurs in both specimens. This shows that the ductile deformation of the polymer matrix is the main reason for the failure of the specimens with a carbon fiber-reinforced polymer matrix, and there will be a new composite structure in the polymer composite. This is because when carbon fiber is pretreated by oxidation, as carbon fibers are oxidized to introduce new functional groups, there will be pits on the carbon fiber surface, which increases surface roughness. When these oxidized carbon fibers are added into the SMP matrix, the chain structure of the SMP will be reacted with the functional groups of the fiber, and because of the existence of the rough surface of the carbon fiber, it is easier for the SMP matrix to be glued onto the surface of the carbon fiber. For the specimens with carbon fiber mass fractions of 10%, 12% and 14%, the tensile sections tended to be coarser and the crack size was larger. This is because, when the amount of carbon fiber is excessive, the dispersion of carbon fiber in the polymer matrix gets worse, which causes a large degree of agglomeration; then, the failure of the specimens is mainly due to the excessive carbon fiber agglomeration.

**Figure 9:**
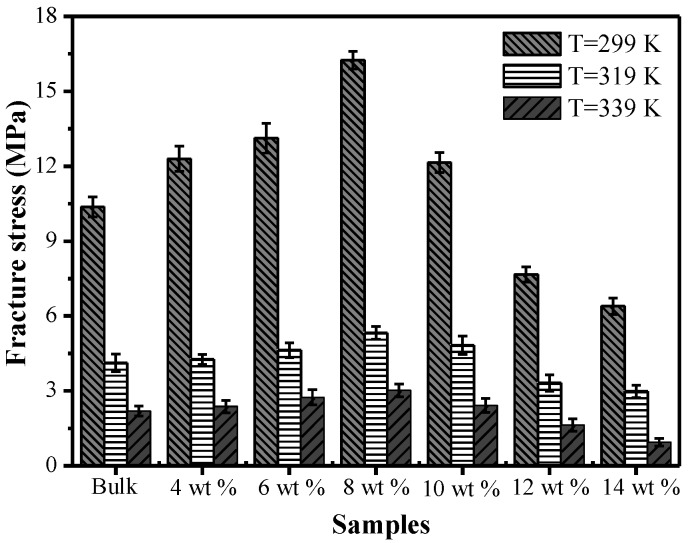
The effects of different chopped carbon fiber contents on fracture stress at three test temperatures of 299 K, 319 K and 339 K.

Figure 9 shows the effect of different chopped carbon fiber content on fracture stress at three test temperatures of 299 K, 319 K and 339 K. In the initial stage, with an increase of the carbon fiber mass fraction, the fracture stress correspondingly increased. When the mass fraction of carbon fiber was higher than 8%, the fracture stress showed a decreasing trend. The maximum fracture stress appeared in the specimen with carbon fiber mass fraction of 8%. At temperatures of 299 K, 319 K and 339 K, the maximum fracture stress of the specimen with a mass fraction of 8% was increased by 56.7%, 29.1% and 31.3%, respectively, compared with that of the bulk specimen. However, the maximum fracture stresses of the specimens with carbon fiber mass fractions of 10%, 12% and 14% were decreased; especially, the maximum fracture stresses of specimens with carbon fiber mass fractions of 12% and 14% were lower than that of the bulk specimen. This is mainly because the dispersibility of carbon fiber is better, and the tensile strength depends on the mass fraction of carbon fiber in the initial stage; with an increment of carbon fiber mass fraction, the dispersibility of carbon fiber gets worse. When the carbon fiber dosage is excessive, the phenomenon of agglomeration occurs, which leads to a decrease of the fracture stress and worse mechanical properties of TPI SMP composites.

**Figure 10:**
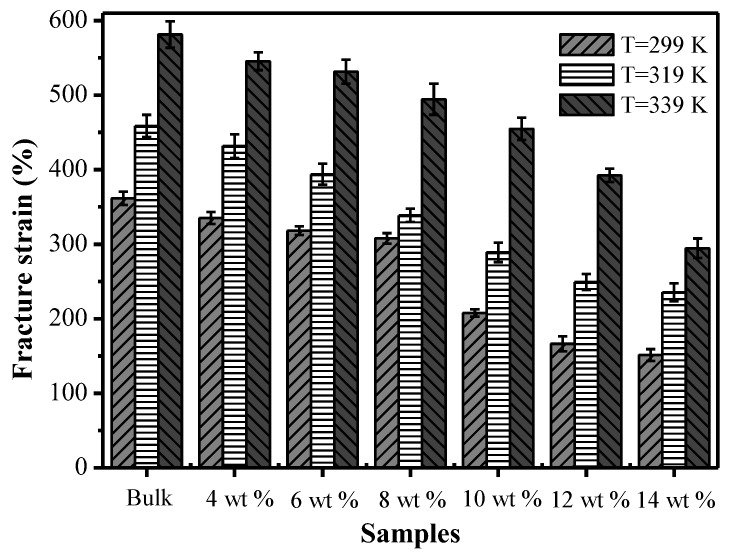
The effects of different chopped carbon fiber contents on fracture strain at three test temperatures of 299 K, 319 K and 339 K.

Figure 10 shows the effects of different chopped carbon fiber mass fractions on the fracture strain at temperatures of 299 K, 319 K and 339 K. At the same temperature, with an increase of the mass fraction of chopped carbon fiber, the fracture strains of specimens decreased. This is mainly because when a specimen is stretched, the molecular chains of the composite material are rearranged along the direction of principle stress, and the composite structure existing in SMPCs will prevent the movement of the molecular chains, and therefore will intensify the difficulty of rearrangement of molecular chains of the SMP and finally cause a reduction of fracture strain of SMP composites. With rising temperature, the fracture strain of SMP composites with the same carbon fiber mass fraction increased constantly. This was caused by Brownian movement of composites during the loading process, and with an increase of temperature, Brownian motion intensifies and the molecular chain movement of the SMP intensifies, making rearrangement of the molecular chains of the SMP easier, thereby causing an increase of the fracture strain of the SMP composites. 

#### 3.4.2. Mechanical Cycle Property

**Figure 11:**
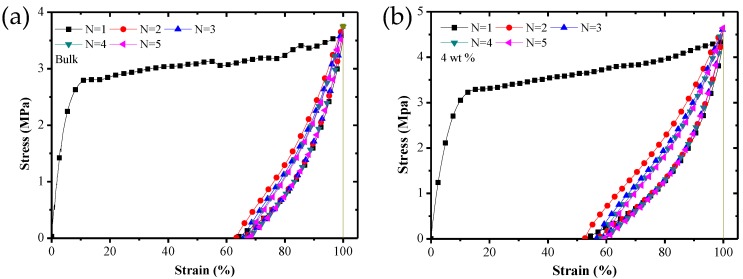

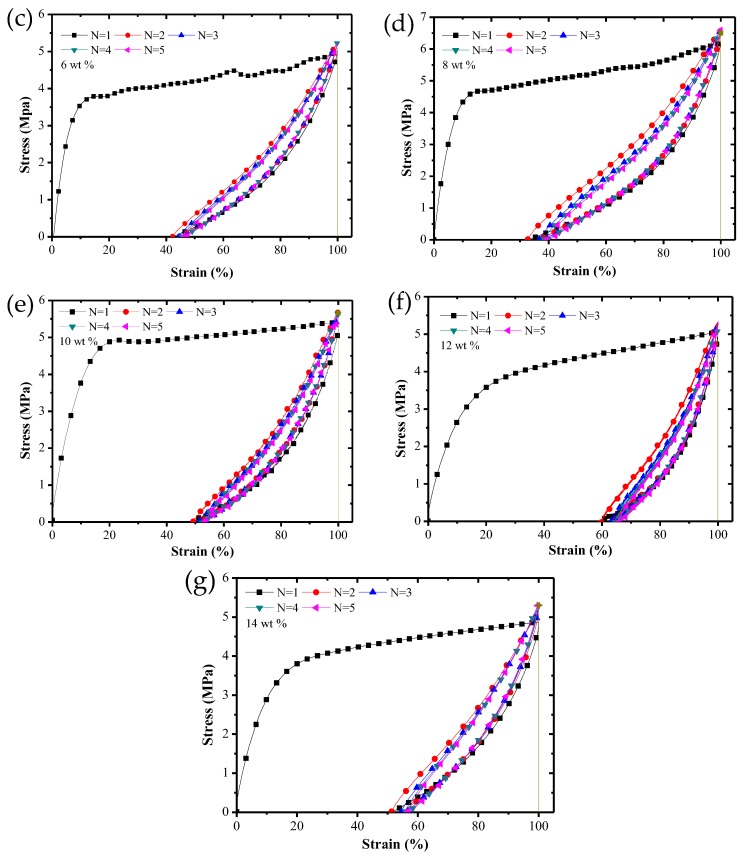
The stress–strain curves of SMP composite specimens with carbon fiber mass fraction of (**a**) bulk, (**b**) 4 wt %, (**c**) 6 wt %, (**d**) 8 wt %, (**e**) 10 wt %, (**f**) 12 wt % and (**g**) 14 wt % under 5 constant strain cycles at room temperature.

Figure 11 shows the stress–strain curves of SMP composites at room temperature under five constant-strain cycles.

It can be seen from Figure 11 that there is a huge hysteresis curve between the first and the second cycles. However, the difference from the second to the fifth cycle is small. This phenomenon is caused by the deformation and failure of parts of composite structures and the special training effect of the SMP in the first cycle.

**Figure 12:**
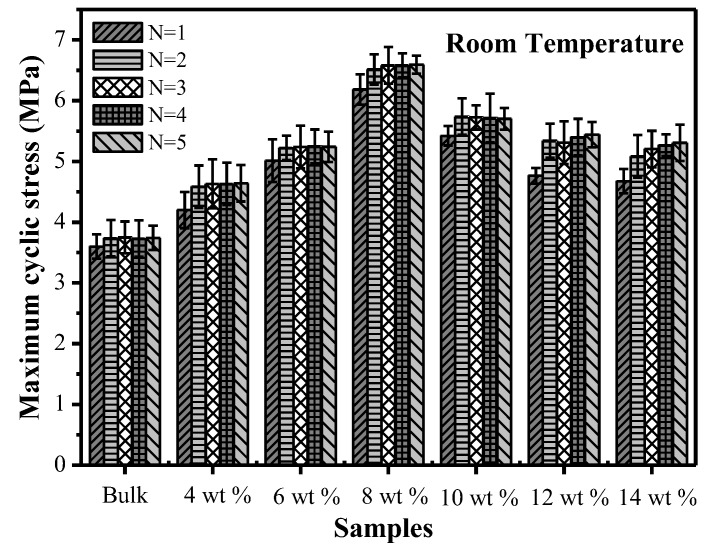
The effect of the number of cycles on the maximum cyclic stresses of SMP composites with different chopped carbon fiber contents.

Figure 12 shows the change of the maximum cyclic stresses of SMPCs with the number of cycles at room temperature. At the starting phase of the cycle, the maximum cyclic stress rapidly increased, and finally tended to be stable as the number of cycles continuously increased. Furthermore, the maximum cyclic stress showed a tendency of firstly increasing and then decreasing as the carbon fiber mass fraction increased. The maximum cyclic stress value of the specimen with a carbon fiber mass fraction of 8% was the highest, which was 76% higher than that of the bulk specimen. The maximum cyclic stresses of specimens with carbon fiber mass fractions of 4%, 6%, 10%, 12% and 14% were higher than that of the bulk specimen by 16%, 38%, 51%, 31% and 29%, respectively. This was mainly caused by the composite structures appearing in SMP composites and the phenomenon of carbon fiber agglomeration. 

**Figure 13:**
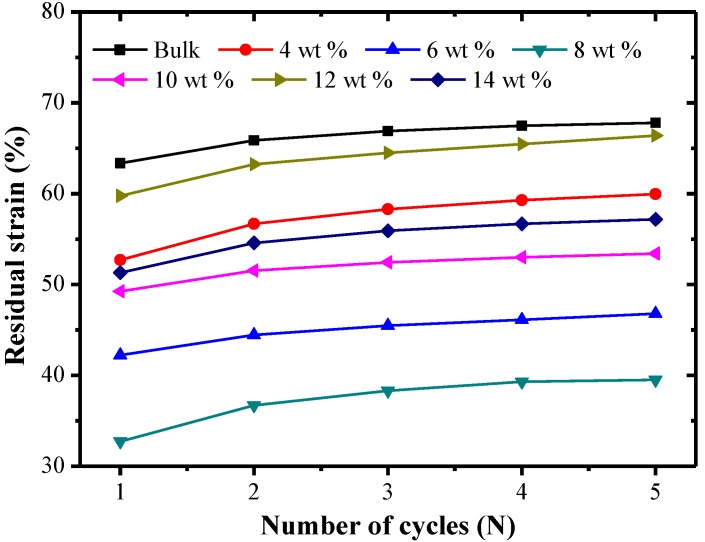
The effect of the number of cycles on the residual strain of SMP composites with different chopped carbon fiber contents.

Figure 13 shows the effect of the number of cycles on the residual strain of SMP composites at room temperature. At the initial phase of the cycle, with an increase of the number of cycles, the residual strain increased firstly and then tended to stabilize. The reason is that when reinforced fibers were added into SMPs, reinforced fibers play the role of resisting deformation and stabilizing the cycle. Furthermore, when the mass fraction of carbon fiber was lower, the residual strain consequently decreased as the carbon fiber mass fraction increased. When the carbon fiber mass fraction exceeded a certain amount, the residual strain had an increasing trend with the increment of carbon fiber mass fraction. The residual strain of the specimen with a carbon fiber mass fraction of 8% was the lowest, which was about 51% of the residual strain of the bulk specimen. The reason is that the recovery force of the composite structure existing in polymer composites is bigger than that in a polymer matrix, so the recovery performance of SMP composites is much better than that of SMP matrices.

#### 3.4.3. Thermal–Mechanical Cycle Property

**Figure 14:**
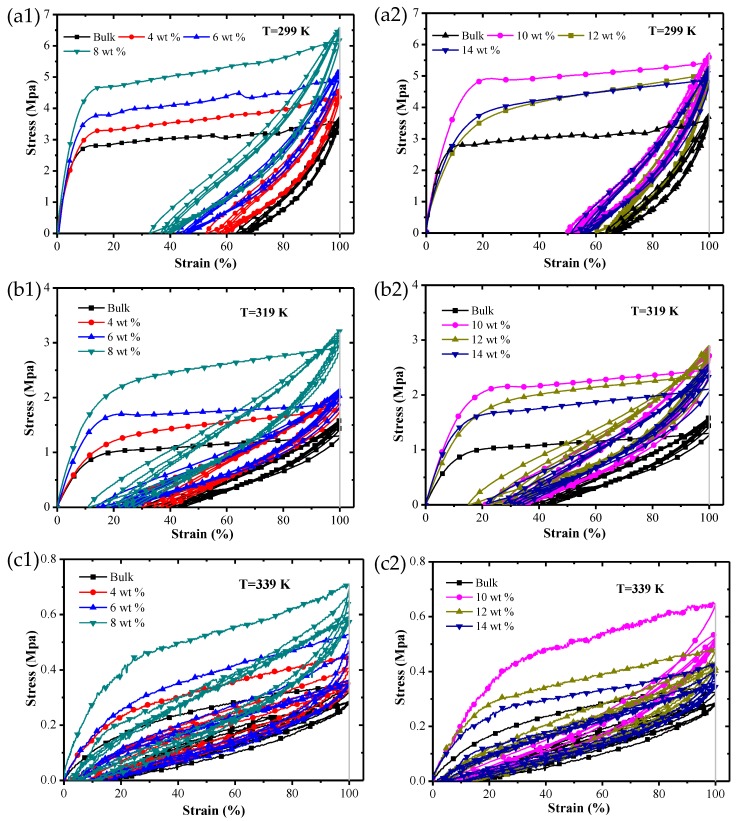
The stress–strain curves of SMP composite specimens with different chopped carbon fiber mass fractions under a constant-strain cyclic loading at three test temperatures: (**a1**,**a2**) 299 K; (**b1**,**b2**) 319 K; (**c1**,**c2**) 339 K.

Figure 14 shows the stress–strain curves of SMP composite specimens with different chopped carbon fiber mass fractions under a constant-strain cyclic loading at three test temperatures: (a) 299 K, (b) 319 K and (c) 339 K.

It can be seen from the figure that at three test temperatures, there was a huge hysteresis curve between the first cycle and the second cycle, but the difference between cyclic curves from the second to the fifth was smaller, and the Young’s modulus of all specimens increased as the number of cycles increased. When the test temperature was 299 K and 319 K, the maximum cyclic stress increased with incrementing cycle number. However, when the test temperature was 339 K, the maximum cyclic stress decreased as the number of cycles increased. This phenomenon is mainly caused by the repositioning of the reinforced fiber and matrix structure. The maximum cyclic stress and residual strain for SMP composites approached stable values as the number of cycles increased, so it was necessary to study the stable cyclic stress and the stable residual strain of SMP composites.

**Figure 15:**
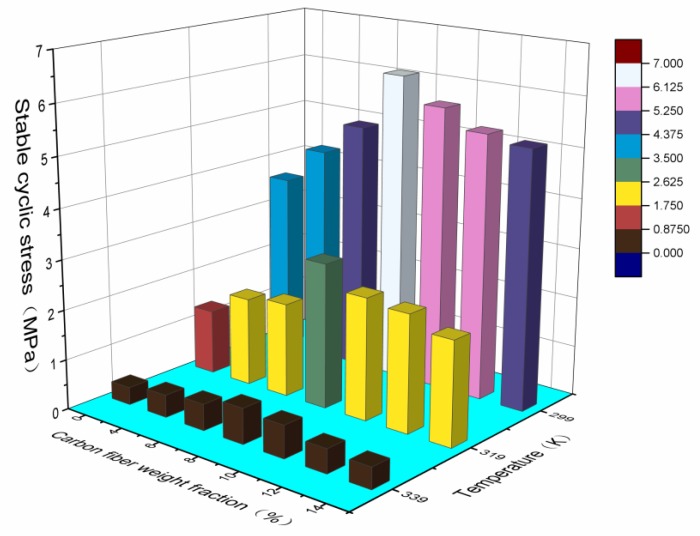
The effect of chopped carbon fiber content on the stable cyclic stress of SMPC specimens at three test temperatures of 299 K, 319 K and 339 K.

Figure 15 shows the effect of carbon fiber content on the stable cyclic stress of SMPC specimens at three test temperatures of 299 K, 319 K and 339 K. When the mass fraction of carbon fiber was lower, the stable cyclic stress increased with the increment of carbon fiber mass fraction. When the mass fraction of carbon fiber reached a certain amount, the stable cyclic stress decreased as the carbon fiber mass fraction increased. The stable cyclic stress value of the specimen with a carbon fiber mass fraction of 8% was the maximum, which was higher than that of the bulk specimen by 72%, 124% and 98%, respectively, at temperatures of 299 K, 319 K and 339 K. This was mainly caused by the composite structure appearing in SMPCs and the occurrence of carbon fiber agglomeration. In addition, the stable cyclic stress of the SMPC decreased with rising temperature. This was because when the temperature is lower than the transition temperature, the tensile modulus of the SMPC is mainly determined by the crystalline state. When the temperature is higher than the transition temperature, it is crystallized and melted, and then, the tensile modulus of SMPCs mainly depends on the mechanical properties of the amorphous state.

**Figure 16:**
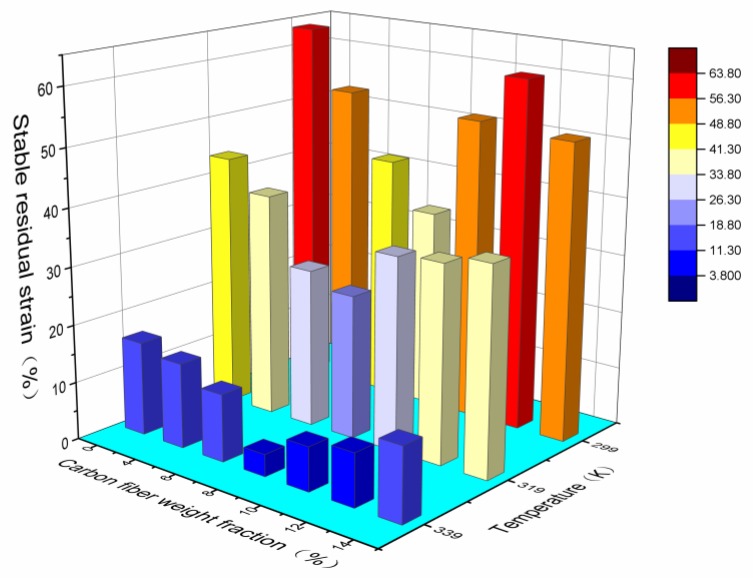
The effect of chopped carbon fiber content on the stable residual strain of SMPC specimens at three test temperatures of 299 K, 319 K and 339 K.

Figure 16 shows the effect of the mass fraction of chopped carbon fiber on the stable residual strain of the SMPC at test temperatures of 299 K, 319 K and 339 K. When the mass fraction of chopped carbon fiber was lower, the stable residual strain of the specimen decreased as the carbon fiber mass fraction increased, and once the carbon fiber content reached a certain amount, the stable residual strain value of the specimen increased slowly with an increase of carbon fiber content. Under all three test temperatures, the residual strain of the SMP composites with a carbon fiber mass fraction of 8% was the lowest, which means that no matter how the temperature changed, the mass fraction of carbon fiber had a direct effect on the residual strain of SMP composites. Furthermore, the stable residual strain of the specimen decreased when the temperature was rising. The reason is that, with rising temperature, the crystallinity area of SMP composites decreased and the recovery ability of SMP composites increased.

### 3.5. Shape Memory Properties

**Figure 17:**
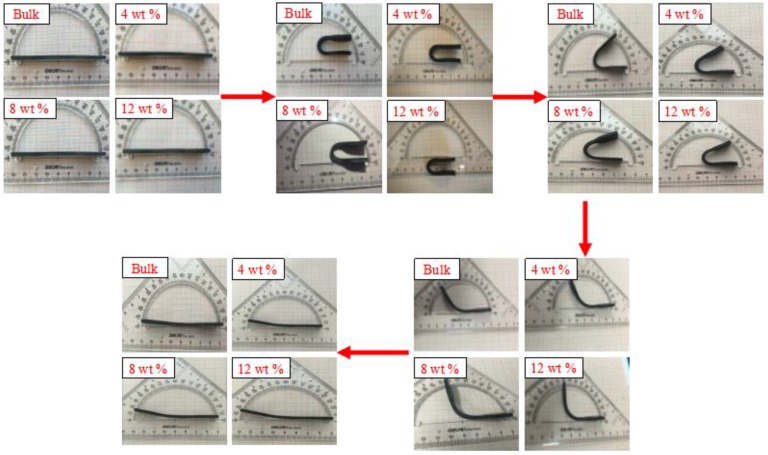
Process for the test of shape memory properties.

Figure 17 shows the pictures of the process for the test of shape memory properties based on the mechanism of thermomechanical deformation of SMPs.

**Figure 18:**
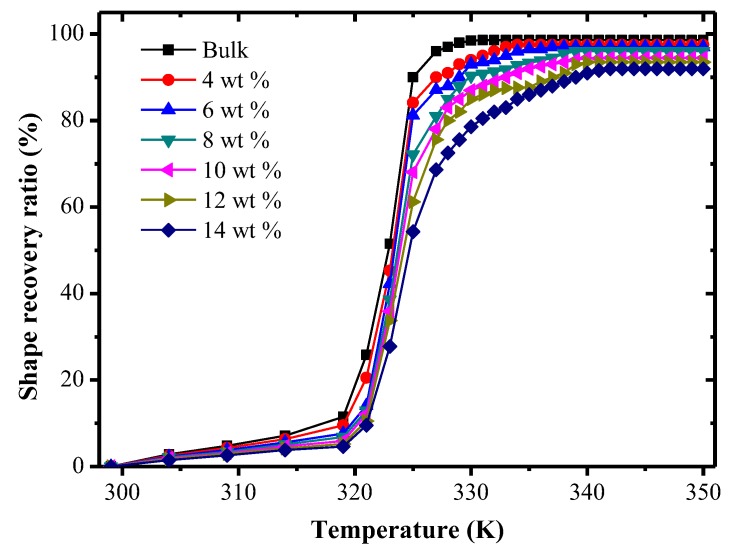
The shape recovery ratio of SMP composites with different chopped carbon fiber contents under different temperatures.

Figure 18 shows the shape recovery ratio of SMP composites with different carbon fiber content under different temperatures. At the initial phase of temperature rising, the shape recovery ratio increased slowly with an increase of the temperature. Additionally, when the temperature rose to 319 K, the shape recovery ratio of specimens with carbon fiber mass fractions of 0%, 4%, 6%, 8%, 10%, 12% and 14% was 11.5%, 9.5%, 7.6%, 6.8%, 5.9%, 5.2% and 4.6%, respectively. After the temperature was higher than the transition temperature, with the temperature continually rising, the shape recovery ratios of all specimens rose rapidly. This is because that for the highly cross-linked structure, the molecular cross-linked network chain is very dense, which has a bigger constraint on the molecular chains. Movement of the cross-linking chain segment needs more energy, and the free space needed for movement of each chain segment is also increased correspondingly. In this way, the macromolecule requires a higher temperature to get enough energy needed for the chain segment, then the frozen stress in chain segment can be released effectively, thereby realizing shape recovery. Although the shape recovery ratio of the bulk specimen was higher than that of other specimens, the shape recovery ratios of all specimens were increased by more than 90%. This shows that the shape recovery performance of TPI SMPCs is excellent.

**Figure 19:**
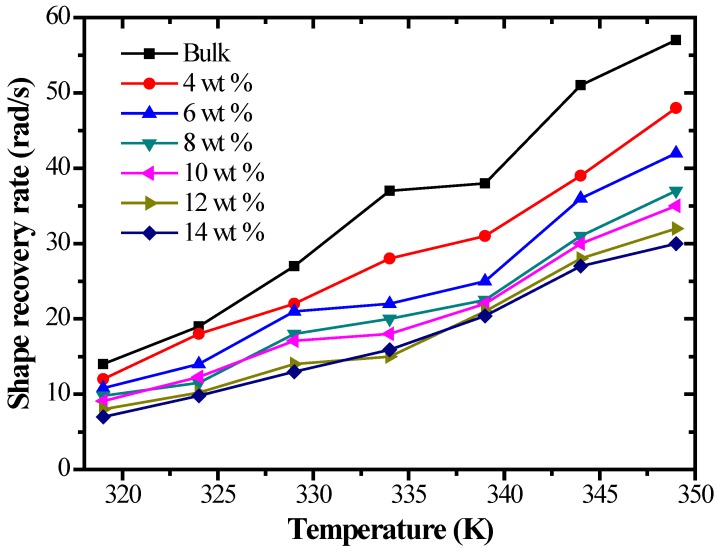
The shape recovery rate of SMP composites with different chopped carbon fiber contents under different temperatures.

Figure 19 shows the shape recovery rate of SMP composites with different carbon fiber contents under different temperatures. The shape recovery rates of all the specimens increased with rising temperature. At the same temperature, with an increase in the carbon fiber content, the shape recovery rate of the specimen decreased. This is because the recovery force of the composite structure in a polymer composite is bigger than that in an SMP matrix. A specimen with more composite structures needs more energy to achieve shape recovery. When the temperature is constant, in order to achieve shape memory recovery, it inevitably needs more time, thereby causing a decrease of the shape recovery rate. 

## 4. Conclusions

In this work, the thermomechanical properties and shape memory properties of TPI shape memory polymer composites with different carbon fiber contents were studied by a DSC test, DMA test, thermal conductivity test, static tensile test, mechanical cycle test, thermodynamic cycling test and shape memory test. The present experimental results show that when the chopped carbon mass fraction fiber was 8%, the TPI shape memory polymer composite has good shape memory properties and the best mechanical properties. This indicates that the 8% carbon fiber mass fraction could be the optimum value for an SMP developed using the proposed manufacturing process.

## Figures and Tables

**Table 1:** The main raw materials formulation.

Material Name	Weight Parts	Material Name	Weight Parts
TPI	100	accelerator CZ	1
light calcium carbonate	10	peptizer (SJ-103)	4
zinc oxide	5	plasticizer (phthalate dioctyl)	2
stearic acid	1	sulfur	1
antioxidant (4010NA)	2	-	-
